# The impact of physical fitness on resilience to modern life stress and the mediating role of general self-efficacy

**DOI:** 10.1007/s00406-021-01338-9

**Published:** 2021-10-07

**Authors:** R. J. Neumann, K. F. Ahrens, B. Kollmann, N. Goldbach, A. Chmitorz, D. Weichert, C. J. Fiebach, M. Wessa, R. Kalisch, K. Lieb, O. Tüscher, M. M. Plichta, A. Reif, S. Matura

**Affiliations:** 1 Department of Psychiatry, Psychosomatic Medicine and Psychotherapy, University Hospital Frankfurt, Goethe-University, Heinrich-Hoffmann-Str. 10, 60528 Frankfurt/Main, Germany; 2 Department of Psychiatry and Psychotherapy, University Medical Center Mainz, Mainz, Germany; 3 Leibniz Institute for Resilience Research (LIR) gGmbH, Mainz, Germany; 4 Faculty of Social Work, Education and Nursing Sciences, Esslingen University of Applied Sciences, Esslingen, Germany; 5 Department of Psychology, Goethe University, Frankfurt, Frankfurt am Main Germany; 6 Brain Imaging Center, Goethe University, Frankfurt am Main, Germany; 7 Department of Clinical Psychology and Neuropsychology, Institute for Psychology, Johannes Gutenberg University Mainz, Mainz, Germany; 8 Neuroimaging Center (NIC), Focus Program Translational Neuroscience (FTN), University Medical Center Mainz, Mainz, Germany

**Keywords:** Physical fitness, Physical activity, Stress resilience, Mental health disorders, Self-efficacy

## Abstract

Substantial evidence shows that physical activity and fitness play a protective role in the development of stress related disorders. However, the beneficial effects of fitness for resilience to modern life stress are not fully understood. Potentially protective effects may be attributed to enhanced resilience via underlying psychosocial mechanisms such as self-efficacy expectations. This study investigated whether physical activity and fitness contribute to prospectively measured resilience and examined the mediating effect of general self-efficacy. 431 initially healthy adults participated in fitness assessments as part of a longitudinal-prospective study, designed to identify mechanisms of resilience. Self-efficacy and habitual activity were assessed in parallel to cardiorespiratory and muscular fitness, which were determined by a submaximal step-test, hand strength and standing long jump test. Resilience was indexed by stressor reactivity: mental health problems in relation to reported life events and daily hassles, monitored quarterly for nine months. Hierarchical linear regression models and bootstrapped mediation analyses were applied. We could show that muscular and self-perceived fitness were positively associated with stress resilience. Extending this finding, the muscular fitness–resilience relationship was partly mediated by self-efficacy expectations. In this context, self-efficacy expectations may act as one underlying psychological mechanism, with complementary benefits for the promotion of mental health. While physical activity and cardiorespiratory fitness did not predict resilience prospectively, we found muscular and self-perceived fitness to be significant prognostic parameters for stress resilience. Although there is still more need to identify specific fitness parameters in light of stress resilience, our study underscores the general relevance of fitness for stress-related disorders prevention.

## Introduction

Being physically inactive ranks among the most important public health problems in modern days, as it has been found to be the fourth-leading risk factor of death worldwide and is associated with increased incidence of mental disorders [1–4]. While prevalence of stress-related mental disorders continues to rise, 25% of European adults are estimated to be insufficiently active which did not change over the past decade [2]. These statistics are startling, given the comprehensive evidence for the potential mental and physical health benefits of physical activity and fitness.

### Physical activity, fitness and mental health

The direct associations between physical activity, physical fitness and mental health are well established. Physically active individuals report better emotional well-being [5] and mood enhancement [6]. Especially, *physical activity* has been suggested as a protective factor against the development of common stress related mental health problems. A large number of clinical, interventional and epidemiological prospective studies have demonstrated a dose-dependent effect of regular exercise or habitual physical activity on depression and anxiety [7–12]. Also *physical fitness*, which may result from the interplay of different factors such as exercise participation, but may also in part be genetically determined [13], has been recognised as an important marker of mental health [14, 15]. Among different health-related fitness components, cardiorespiratory fitness (CRF) has most frequently been associated with lower rates of stress related mental health problems such as depression and burnout [16–19]. In a recent meta-analysis of prospective cohort studies Kandola et al. [20] suggested a dose–response relationship between CRF and the risk of common mental health disorders*.* Although less well-established than CRF, muscular fitness has increasingly been recognised as an important physical parameter. Muscular strength has been positively associated with mental resources, health-related quality of life in adolescents and aged people [14, 21], and inversely associated with the development of depressive symptoms and frailty [22–24]. While Kettunen et al. [21] concluded that both, cardiorespiratory and muscular fitness are associated with mental resources in men, the additional value of muscular fitness remains unclarified. Åvitsland et al. [25] indicated that muscular fitness is independently associated with mental health problems, however, when controlling for CRF, the effect vanished. Nonetheless, as recently suggested by Tacci et al. [17], objective fitness measurements and the onset of mental health problems are an emerging field. Based on the lack of methodological comparable investigations and data on both, activity levels and objective fitness measures, there is a substantial need for population-based studies focussing on the association between fitness components and future risk of adverse mental health outcomes.

### Physical activity and fitness as stress-buffers and resilience factors

While the direct relationship between physical activity, fitness and common mental health disorders has been widely recognised, the amount of stress individuals are facing is rarely considered. In resilience research the focus lies on preventive factors and mechanisms that have the potential to mitigate undesirable consequences of stress and promote mental health in the face of stressor exposure. Lately resilience has increasingly been conceptualised as the outcome of a dynamic process of adaptation in the face of adversity and changing demands [26–28]. Thus individuals with high resilience are less likely to develop mental health problems than expected in proportion to the accumulated stressor load [29]. A few cross-sectional and longitudinal field studies addressed the protective, stress-buffering effects of exercise and fitness, which have both been associated with a better capacity to cope with chronic stress, less health-complaints, and higher self-perceived resilience [18, 21, 30–32]. Interestingly, according to Klaperski, Seelig, and Fuchs [33], in longitudinal analyses, chronic exercise participation was capable to buffer moderate intense chronic stress. But the authors found no evidence for this stress-buffering function of physical activity against the effects of acute stress in the cross sectional design. A recent study by Schilling et al. [34] addressed the potential stress buffering effect of CRF in a real-life study on police officers and indicated lowered physiological stress reactivity to acute work stress in police officers with higher levels of CRF. Still, the literature offers insufficient evidence concerning the protective role of physical fitness to everyday life stressor exposure or modern life stress, including daily hassles and life events.

### Psychological mechanism: general self-efficacy

Insight into the mediating pathways is essential to understand the mechanisms underlying the beneficial effects of physical fitness on stress coping. With regard to cognitive appraisal, the concept of self-efficacy, as initially introduced by Bandura’s social cognitive theory, refers to the belief of one’s capabilities to perform properly in challenging situations [35–37]. It has been proposed as a resistance resource within the cognitive appraisal process, which is known to be crucial for the regulation of stressful, potentially traumatising demands [38, 39]. The stress regulatory capacities are used as a possible explanation in research proposing that efficacy expectations buffer the negative effects of daily stress or life events on mental health problems [40–44]. More recently, self-efficacy has also been postulated as an essential resilience mechanism in the positive appraisal style theory of resilience (PASTOR) [45]. Earlier theoretical models state that changes in physical activity and fitness can act as a mastery or efficacy experience in the physical domain [46, 47]. This experience may even generalise to a broader physical self-concept and, consequently, to enhanced global self-esteem and psychological wellbeing. Interestingly, self-efficacy has been shown to be both, a determinant as well as a consequence of physical activity [47]. Nonetheless, to our knowledge, the mediating effect of self-efficacy on the relation between physical activity and fitness on resilience to stress has not systematically been examined yet. This study is motivated by the hypothesis that physical activity and fitness alter stress perceptions, i.e., the way we interpret stressful situations, and by this play a crucial role in coping with everyday stress.

### Study aims

Our primary aim is to extend earlier findings of a “stress-buffering effect” of activity and fitness, and to examine, whether health-related fitness components CRF, muscular strength, self-perceived fitness and physical activity predict mental health outcomes in the face of modern life stress, hence resilience to stress. Our second objective is to investigate whether the effects of physical fitness and activity on resilience are mediated by self-efficacy as a cognitive mechanism. We postulate that self-efficacy partly explains the relation between physical activity, fitness and resilience to stress. To investigate the association between physical fitness, self-efficacy and resilience we analyse a sub-sample from the *LO*ngitudinal *R*esilience *A*ssessment (LORA) study,—a longitudinal prospective study to identify various mechanisms of resilience.

## Method

### Study design and participants

The LORA study is a population-based, multi-center cohort study. Data collection for baseline assessment at the study sites Frankfurt and Mainz started in 2017 and continued until 2019. Planned longitudinal assessment will be ongoing for at least 4.5 years [48]. Participants from the Rhine-Main Area were recruited via online or printed public advertisements at the local universities, medical centres, libraries, shops and gyms and the projects webpage (https://lora-studie.de/). After agreement to study participation, study eligibility was tested via a structured telephone interview. The inclusion criteria were age 18 to 50 years, having normal or corrected vision and sufficient knowledge of the German language. The exclusion criteria were lifetime diagnosis of schizophrenia or bipolar disorder, organic mental disorders or substance dependence syndromes, as well as any other current severe axis-I disorder or medical conditions. Participants with known learning disabilities, serious neurological disorders (e. g., tumours in the central nervous system), or participants who had taken part in a drug trial in the previous six months were also excluded.

**Fig. 1:**
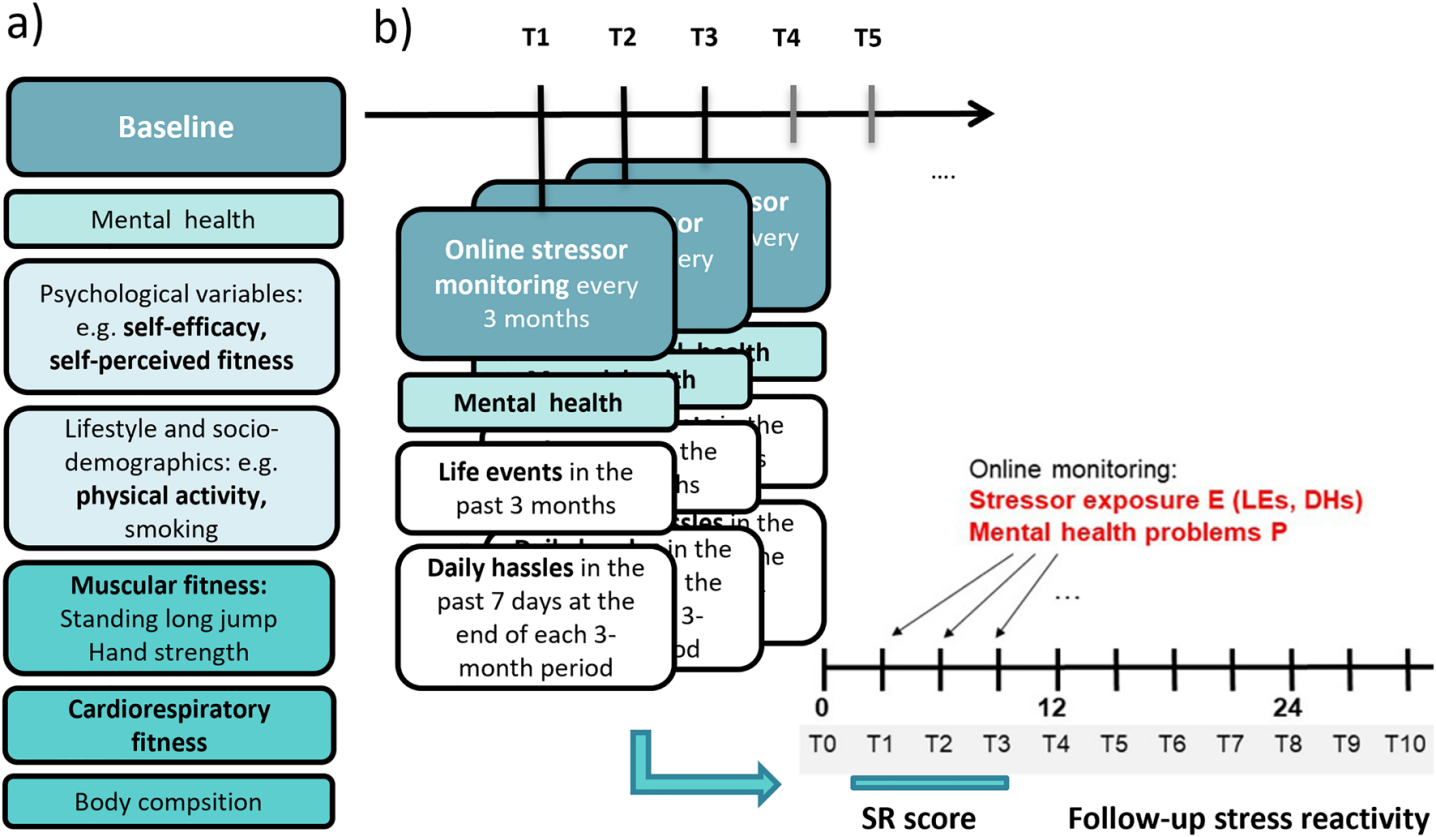
Adapted LORA study design from Chmitorz et al., [48] with **a** selected baseline measurements: Physical fitness components, physical activity, self-perceived fitness and general-self efficacy and **b** the follow-up interim stressor-monitorings in 3-months intervals. Follow-up measurements are used to build up the stressor reactivity score (SR score) as an outcome

In total, 1191 healthy were enrolled, of which 472 subjects fully completed a fitness test at baseline assessment (T0). After participants were found eligible, they were invited for an extensive baseline assessment (T0), where they received further detailed information about study participation and were asked to give informed consent. A clinical interview and anthropometric and fitness measures were conducted. After the in-house assessment participants were introduced to the online database system (secuTrial© electronic data capture system, www.secutrial.com), which adheres to the Guidelines for Good Clinical Practice (GCP). Herein participants were asked to complete the questionnaires on socio-demographics, mental health, life history, psychological, and lifestyle-related variables including proposed resilience factors within a week following the visit. For longitudinal assessment of mental health and stressor exposure, an interim online stressor monitoring was applied every three months, using the same online data base system. In this study, relevant fitness and health measurements at baseline (T0; Fig. 1a) and stressor monitoring of the first nine months (T1-T3; Fig. 1b) were evaluated. Figure 1 displays the adapted study design.

### Instruments

Physical fitness at baseline (Fig. 1a) was assessed according to the standard procedures and recommendations for assessing physical activity levels at population level (ALPHA [49]).

*Cardiorespiratory fitness* (CRF) was measured by the Chester Step Test (CST) to predict maximal oxygen uptake (VO2max) [50]. The CST is a sub-maximal, multistage test, where the participants step on and of a 30-cm step on timed metronome rhythms. For a detailed description of the Chester Step Test see Sykes [50]. For reference, average aerobic capacity (mlsO2/kg/min) for women between 20 and 29 years are 35–43 mls02kg/min and for men in the same age range 32–38 mls02kg/min [50].

A composite measure for *muscular fitness/strength* was built upon handgrip strength (representative for upper body isometric strength) and a standing long jump (representative for lower body strength). Maximum handgrip strength [51] is a widely used test for assessing muscular fitness in adults and was measured by using a handgrip dynamometer (T.K.K.5001, Grip-A; Takei, Japan). For the assessment, participants were asked to stand with their extended arms by their side and, on command, squeeze the handgrip continuously as hard as possible. To determine the full potential of handgrip strength, the optimal grip-span of the dynamometer was accustomed according to sex and hand size. Alternating between left and right hand the test was performed twice per hand. The maximum score for each hand, expressed in kg, was recorded and the highest score for the stronger hand was used for the analyses [52]. Whereas, norms highly depend on age, sex or the instrument used, earlier studies found mean values of 39.1 ± 12.8 kg among healthy adults across the life span [53, 54]. Furthermore, for the assessment of lower body explosive muscular strength, the participants completed a standing long jump [55]. Therefore, they were instructed to jump from a starting line, to push off vigorously and to jump as far as possible. Participants had to land on both feet and stay upright. Then the distance between the first heel-mark and the take-off-line was recorded in centimeters and the best attempt was considered for muscular strength measurement [14, 56].

Self-perceived fitness was assessed using the International fitness scale (IFIS [57]), developed by the PROFITH research group, Granada, Spain, to rate participants according to their overall physical fitness by the main physical fitness components. The IFIS is a self-reporting questionnaire composed of five questions on participants’ perceived physical fitness: cardiorespiratory fitness, muscular fitness, speed–agility, flexibility and overall fitness in comparison with others' physical fitness. For an overall perceived fitness rating, all five questions were summed up to one fitness score.

The International physical activity questionnaire (IPAQ [58]) was used to assess four dimensions of physical activity: occupation, transportation, household activities, and leisure-time, as well as sedentary behaviour in a typical week. The self-report questionnaire asked participants to recall activities for each of the seven preceding days that measure the time spent engaged in walking, moderate activity (e.g., carrying light loads, bicycling or easy swimming), vigorous intensity activity (hard physical effort e.g., lifting, aerobics, fast running) and sedentary behaviour. The sum of weekly metabolic equivalent (MET)-minutes per week was used as a continuous activity indicator of the four categories. For analyses the weighted MET-minutes per week were summed up across walking, moderate and vigorous intensity activities to produce a weighted estimate of total physical activity per week. The IPAQ is a valid and reliable surveillance tool to monitor physical activity levels [59]. High levels of physical activity are characterised by at least 3000 MET minutes per week [60].

For anthropometric measures weight was taken using a calibrated electronic scale (Seca, Birmingham, UK) with an accuracy of 0.1 kg and height with a stadiometer (Seca) with an accuracy of 0.1 cm. Body Mass Index (BMI) was calculated in kg/height in m^2^.

The General Self-Efficacy Scale (GSE; [61, 62]) was applied to measure an estimate of general perceived self-efficacy across 10 items, which were rated on a 4-point Likert-scale (from ‘not at all true’ to ‘exactly true’). The GSE covers judgments concerning causal and control beliefs, such as the extent to which one attributes the successful handling of difficult situations to one’s own competence.

Resilience to stress, as displayed in Fig. 1b, is operationalized as mental health status corrected for stressor exposure, which indicates individual reactivity of mental health to stressors: *stressor reactivity score (SR score).* Participants were asked to report on their experienced stressor exposure every three months over a nine-month period, including macro-stressors (critical life events) and micro-stressors (daily hassles). Exposure to critical life events was collected retrospectively, using an adapted German version of a standard life events (LE) checklist from Canli et al. [63] The questionnaire lists critical or major life events and incidents of potentially traumatising events. The adapted German version contains 27 items, for which participants indicated whether the respective event occurred within the previous three months. For a life event sum score, occurrences of events were summed up. Regarding micro-stressors, chronic stressors and daily hassles (DH) were assessed using the Mainz Inventory of Microstressors (MIMIS), which was recently developed and validated by Chmitorz et al. [64]. Here, participants are asked to retrospectively report the number of days the stressors occurred from a list of 58 DH (ranging from 1 to 7 days) within the past seven days, including the day of assessment. For analyses, DH were calculated by multiplying the total amount of all hassles reported in the past week with the reported number of days on which a hassle occurred (range 0–58 × 7 days = 406). For the assessment of general health status, the German version of the General Health Questionaire-28 (GHQ-28; [65, 66]) was used. The GHQ-28 is a 28-item measure of emotional distress and rates participant’s subjectively reported health over the last couple of weeks on a four-point Likert scale ranging from 0 (least symptomatic answer) to 3 (most symptomatic answer) with the possible total score ranging from 0 to 84. Goldberg (1978 [65]) suggests a threshold for psychiatric distress of a total sum score of 23/24. Items are assessed on four scales, i.e., somatic symptoms, anxiety/sleeplessness, social dysfunction, and severe depressive symptoms.

### Statistical analysis

For all statistical analyses IBM SPSS Statistics version 25 for Windows (Armonk, NY, USA) was used. Prior to analyses raw data was tested for normality distribution of residuals, errors, plausibility, excessive missing cases, outliers and studentized deleted residuals, resulting in *N* = 431 cases being included into statistical analyses. Spearman’s rank correlations were performed to reveal the differences in mean demographics (sex, age, BMI, income, education level) and independent variables (IV). Accordingly, covariates were included in the analysis.

For the quantification of resilience as an outcome, in accordance with Kalisch et al. [29], we used a residualization-based calculation of stressor reactivity (SR score) during a timeframe of nine months (T1–T3, Fig. 1b.). The SR score was used as the outcome variable for hypothesis testing. Participants of the full study sample, who provided a minimum of one complete 3-monthly monitoring, were included in the analysis (*N* = 1078). A high SR score implies low resilience and *high stressor reactivity,* meaning more mental health problems in proportion to the stressor load. A low SR score indicates high resilience and *low stressor reactivity*, thus relatively low-mental health problems in response to stress.

Before main analyses, all fitness components were transformed into standardised values (Z-scores) to relate them to relative fitness levels. Z-scores for the standing long jump and hand strength were combined to a composite mean score for muscular strength.

For main hypotheses testing a hierarchical multiple regression model was conducted to examine whether physical fitness and activity (independent predictor variables) predict stress reactivity over a 9-months timeframe (dependent variable). Sex, age and BMI were entered at stage one of the regression to control for these influencing variables. Physical fitness and activity were entered at stage two with forced entry to determine the true correlation between fitness and stress reactivity controlling for the effect of potential influencing factors.

To test whether the association between the fitness components and stress reactivity was mediated by general self-efficacy, mediation analyses were performed using the PROCESS macro by Hayes [67]. Unstandardized path coefficients for total, direct, and indirect effects were estimated by means of ordinary least squares (OLS) regression analyses. Sex, age and BMI were entered as covariates. Bootstrapping with 5000 samples together with heteroscedasticity consistent standard errors [68] were fitted to compute the confidence intervals and inferential statistics. Effects were deemed significant when the confidence interval did not include zero. The coefficients (a, b and c’) represent the fully standardised regression coefficients. A *p* value of 0.05 (two-tailed) or smaller determines statistical significance.

## Results

### Participant characteristics, correlations, and stress reactivity calculation

Descriptive statistics for all relevant variables over the whole sample, consisting of 431 participants with complete data for fitness measures, are displayed in Table 1. GHQ mean score significantly increased (i.e., average mental health worsened) from the baseline assessment 16.33 (± 7.54) to an average of 20.51 (± 7.70) over the nine months (T1–T3). Quantification of stressor reactivity score (SR score; based on linear regression modelling across T1 to T3) indicated a significant linear positive relationship between combined stressor exposure and mental health problems (*R* = 0.41, *p* < 0.001, *N* = 1078).

**Table 1:** Participants’ sociodemographic and anthropometric characteristics and tested variables

	Total sample mean ± SD / frequency	Range
Age	27.15 ± 6.85	18–50
Sex		
♀ ♂	274 (63.6%) 157 (36.4%)	
Highest educational achievement (T0)		
School-leaving certificate Certificate of Secondary Education School leaving examination Completed vocational training University degree	1 (0.2%) 8 (1.9%) 188 (44.3%) 53 (12.5%) 176 (41.0%)	
Employment (T0)		
Full-time Part-time No employment Currently obtaining an education	109 (25.8%) 42 (9.9%) 10 (2.3%) 262 (61.9%)	
Marital status (T0)		
Non-married Married Separated/divorced	359 (85.1%) 59 (14.0%) 4 (0.9%)	
Smoking (yes; no) (T0)	46 (10.7%); 385 (89.3%)	
Body mass index (BMI) (T0)	23.29 ± 3.39	
Handgrip strength (kg) (T0)	35.85 ± 10.63	0–100
Standing long jump (cm) (T0)	162.69 ± 34.05	
Aerobic capacity Vo2max (O2/kg/min) (T0)	46.71 ± 10.41	
Total physical activity (MET) per week (IPAQ) (T0)	4269.09 ± 3011.82	
Self-perceived fitness (IFIS) (T0)	18.34 ± 3.06	0–25
General self-efficacy (GSE) (T0)	30.13 ± 4.02	0–40
GHQ-28 baseline (T0)	16.41 ± 7.54	0–84; cut off 23/24
GHQ-28 (mean T1-T3)	20.51 ± 7.70	0–84
Number of Life Events (past 9 months, mean T1–T3)	2.01 ± 1.36	0–27
Number of Daily Hassles (per week, mean T1–T3)	61.44 ± 25.22	0–58 × 7 days = 406

*N* = 431

T0 indicate the baseline data

T1–T3 portray the follow-up data which are used to calculate the SR score, including mental health problems and stressors within the upcoming 9 months

Percentage based on valid data; mean and standard deviation based on all obtained data.

Table 2 depicts the partial intercorrelations of all tested variables, controlled for relevant covariates sex, age and BMI. GHQ at baseline was included as control variable. The main outcome, stressor reactivity over T1 to T3, where a lower value reflects greater resilience to stress, was negatively related to baseline (T0) muscular strength and self-perceived fitness.

**Table 2:** Intercorrelations of all tested variables

Variable	SR	PA	CRF	MS	SPF	GHQ-28		GSE
SR score (T1–T3)								
PA	− 0.03							
CRF	− 0.02	0.18**						
MS	− 0.15*	0.03	0.11					
SPF	− 0.19**	0.26***	0.29***	0.35***				
GHQ-28	0.32***	0.04	0.03	− 0.07	− 0.17**			
GSE	− 0.14	0.05	− 0.07	0.13	0.22***	− 0.26***		

*SR score* stressor reactivity across T1 − T3, baseline variables: *PA* physical activity, *CRF* cardiorespiratory fitness, *MS* muscular strength, *SPF* self-perceived fitness, *GHQ-28* General Health Questionnaire, *GSE* general self-efficacy

*N* = 431

Bonferoni–Holm corrected partial correlations controlled for sex, age, BMI are presented; adjusted *p* values due to Bonferroni–Holm method *p < 0.05, **p < 0.01, ***p < 0.001.

### Predicting resilience from activity and fitness

The two-stage hierarchical regression model with stressor reactivity as the dependent variable is shown in Table 3. Relevant covariates (sex, age, BMI) were entered at stage one of the regression analysis to control for confounding demographic effects. The four baseline variables for activity and fitness were simultaneously entered at stage two. The results revealed that at stage one the demographic variables contributed significantly to the regression model, *F*(3, 427) = 5.26, *p* = 0.001 and accounted for 2.9% of the variation in stressor reactivity, with sex and age being significant. Introducing the four additional independent fitness variables at stage two, accounted significantly for 6.2% of the variation of stressor reactivity, *F*(4,423) = 5.10, *p* = 0.001. Muscular strength (*B* =  − 0.14, *p* =  < 0.01, 95% CI [− 0.28, − 0.04]) and self-perceived fitness (*B* =  − 0.15, *p* = 0.01, 95% CI [− 0.25, − 0.05]) were both significantly negatively related to stressor reactivity. However, neither CRF nor physical activity score were significant predictors of stressor reactivity. Our results suggest that higher muscular strength and higher self-perceived fitness are significantly related with higher resilience to stress.

**Table 3:** Summary of hierarchical regression analysis with Stressor reactivity (SR) score as dependent variable

Variable	B (95% Cl)	β	*t*	*∆R*^*2*^	*p* value
Step 1				0.029	< 0.01
Sex	0.32 [− 0.13, 0.51]	0.17	3.28		0.001
Age	− 0.12 [− 0.23, 0.18]	− 0.12	− 2.31		0.02
BMI	0.09 [− 0.03, 0.21]	0.08	1.40		0.16
Step 2				0.062	< 0.01
Sex	0.08 [− 0.20, 0.35]	0.04	0.54		0.59
Age	− 0.13 [− 0.24, − 0.03]	− 0.12	− 2.44		0.02
BMI	0.07 [− 0.05, 0.19]	0.06	1.10		0.27
Muscular strength	− 0.14 [− 0.28, − 0.04]	− 0.16	− 2.02		0.04
Self-perceived fitness	− 0.15 [− 0.25, − 0.05]	− 0.15	− 2.94		< 0.01
CRF	0.04 [− 0.48, 0.13]	0.05	0.20		0.36
Physical activity	0.01 [− 0.09, 0.11]	0.01	0.27		0.79

*N* = 431

95% bias corrected and accelerated confidence intervals reported in parentheses.

### Mediation by self-efficacy on resilience to stress

**Fig. 2:**
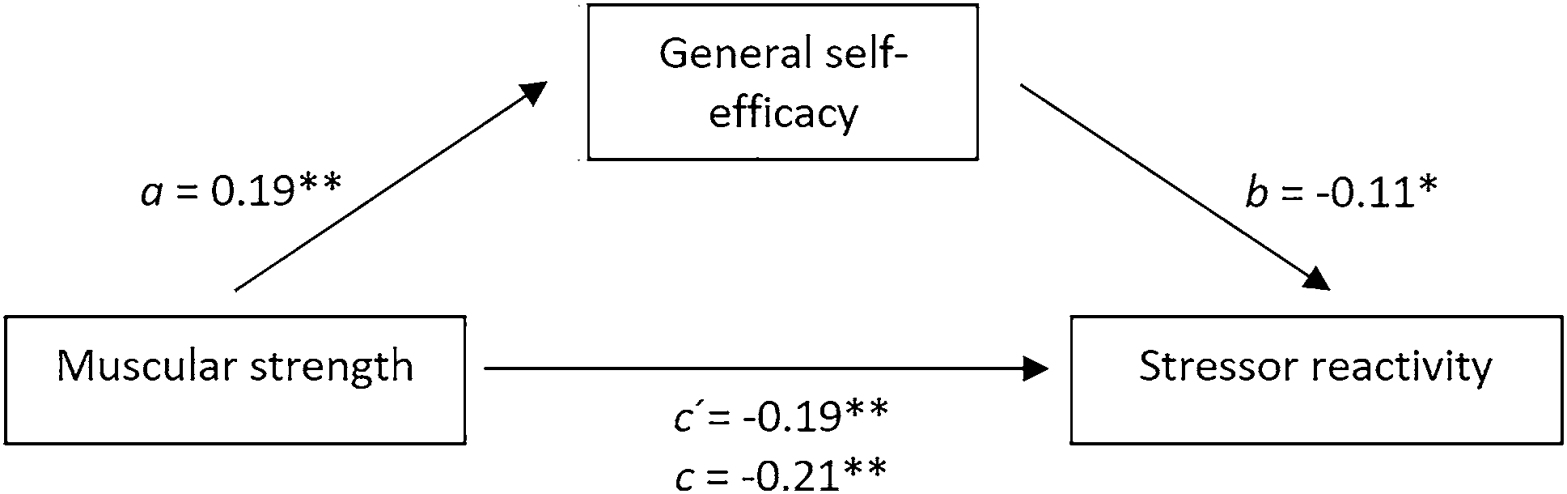
Conceptual and statistical diagram of the mediation model for the direct and indirect effects of muscular strength on stress reactivity. Regression coefficients (fully standardised): **a** effect of muscular strength on general self-efficacy, **b** effect of general self-efficacy on stressor reactivity, **c’** direct effect of muscular strength on stressor reactivity, **c** total effect of muscular strength, general self-efficacy on stressor reactivity. **p < 0.01. *p < 0.05

Two simple bootstrapped mediations analyses were performed to examine whether the direct paths between muscular fitness (mediation model 1) or self-perceived fitness (mediation model 2) and stressor reactivity would be mediated by general self-efficacy. Within the first mediation model all regression coefficients were statistically significant (see Fig. 2). In accordance with primary regressions, the direct, inverse effect of muscular strength on stressor reactivity remained significant, path c’: *b* =  − 0.19, *p* = 0.004, 95% CI [− 0.32, − 0.06]. Entering the mediator into the model resulted in muscular strength being a significant predictor for general self-efficacy, path a: *b* = 0.19, *p* = 0.009, 95% CI [0.04, 0.34], which in turn significantly predicted stressor reactivity, path b: *b* = − 0.11, *p* = 0.014, 95% CI [− 0.20, − 0.02]. The results show that the relationship between muscular strength and stressor reactivity was partially mediated by general self-efficacy with a statistically significant bootstrapped standardised indirect effect path c: *ab* =  − 0.02, 95%BCa CI [− 0.05, − 0.001], with *R*^*2*^ indicating that the model explains 5.8% of the variance in stress reactivity. This indicates that the negative effect of muscular strength on stressor reactivity can partly be explained via general self-efficacy. The effect size points towards a small mediation effect.

Within the second mediation model, a significant direct, inverse effect of self-perceived fitness on stressor reactivity was observed, path c’: *b* = − 0.15, *p* = 0.002, 95% CI [− 0.25, − 0.05], and self-perceived fitness was significantly associated with general self-efficacy, path a: *b* = 0.24, *p* < 0.001, 95% CI [0.11, 0.36]. General self-efficacy significantly predicted stressor reactivity, path b: *b* =  − 0.09, *p* = 0.04, 95% CI [− 0.18, − 0.005]. The bootstrapped indirect effect for self-efficacy was significant, path c: *ab* =  − 0.02. However, the confidence interval range contains zero, 95%BCa CI [− 0.04, 0.001]. Therefore, there is no mediating effect of self-efficacy on the association between self-perceived fitness and resilience to stress.

## Discussion

### Physical fitness predicts resilience to stress

The main findings of this longitudinal study support the assumption of physical fitness to be a predictor of mental health and extend existing research by focussing on resilience to modern life stress as an outcome [21, 69, 70]. The results show that muscular and self-perceived fitness are positively associated with resilience to stress, indicated as low symptomatic stressor reactivity over several months. Generally, the inverse association between muscular strength and mental health problems is in line with earlier investigations that target the predictive value of fitness for mental health resources, quality of life or the development of symptoms of depression and anxiety [14, 21, 22]. Still, until now there is only limited evidence for the specific effects of muscular and self-perceived fitness in relation to stress reactivity in natural settings [18, 21, 34, 71]. Ortega et al. [72] compared the predictive value of self-perceived fitness and objective measures of fitness, here CRF, concerning the risk for developing physical illness. They provided evidence that self-perceived fitness was a valid and reliable instrument to measure objective fitness, and that self-perceived fitness and cardiovascular disease prognosis are strongly associated.

Neither self-reported physical activity nor CRF predicted resilience to stress. The finding, that objective measures outperform habitual physical activity is in accordance with Baumeister et al. [73]. In a prospective, population-based study they showed that leisure-time, work- and sport-based physical activities were not significantly associated with common mental health disorders. Interestingly, objective fitness measures, here greater CRF, were associated with a lower incidence of depression and clinical anxiety. At the same time, the stress buffering, positive mental health effect of actual physical activity has been often reported. One explanation for the current finding could be that objectively assessed fitness provides a more stable health marker over time than self-reported physical activity. Particularly muscular strength may reliably mirror real participation in exercise and sports and therefore the state of the physiological system [74]. Even though self-reports, e.g., the physical activity questionnaire (IPAQ) capture a broad range of physical activities, self-report measures are often criticised to be subject to attentional biases or may rather fluctuate over time, depend on mood, or season [75, 76]. In addition, substantial discrepancies between subjective and objective (e.g., accelerometery) measures of physical activity, typically recording activity over a week, have previously been reported. Real-time measurement of activity and accelerometery may be a more accurate method for actual activity levels [75, 77]. Moreover, Lee et al. [78] compared data on physical activity levels and objective fitness measures. Herein, the described associations between activity and objective measures were gauged as poor and did not reach objective standards. Our results depict a similar picture, as the reported activity levels did not correlate with our objective fitness measures. Although we did not find a direct relationship between self-reported physical activities and fitness measures, regular health-related activities might, nonetheless, partly explain the positive link between fitness and resilience to stress. Therefore, future studies should still integrate measures on activity and fitness and examine whether the interplay between objectively measured physical activity (e.g., using accelerometers) and fitness together can predict resilience to stress and mental health.

With this study we could not replicate earlier findings of a positive association between CRF and mental health [20, 21] or between CRF and resilience to stress [34, 79]. On the one hand, the interpretation should consider that our sample depicts a rather low variance in the measure of maximal oxygen capacity. Up to 75% of the participants scored above the average [50], indicating an overall high performance on the test. This might partly be an explanation for the low predictive power of CRF for resilience outcomes in the long-term. For future studies it is necessary to include participants with larger variance in performance levels, including low, medium and high performers. On the other, preceding studies used self-assessed resilience rather than a stressor-normalised resilience score which may also account the lack of a positive association between CRF or PA with resilience.

### Self-efficacy as a mediating mechanism

To better clarify the still unresolved mechanisms underlying the beneficial effect of physical fitness on resilience, the mediating role of general self-efficacy expectations between fitness and resilience was analysed. Since, only muscular and self-perceived fitness were significantly related to resilience, we restricted mediation analyses to these two fitness components. Our hypothesis could be confirmed for muscular fitness, as the results showed that the relationship between muscular strength and resilience was partially mediated via general-self efficacy. Almost 20% of the variance within the effect of muscular fitness on resilience could be explained by self-efficacy expectations. However, for self-perceived fitness the mediation through self-efficacy did not reach significance, when introducing sex age and BMI as covariates. According to these findings, we assume that the positive effect of the actual (muscular) fitness levels is more likely to be explained by subsequent cognitive self-efficacy expectations*.* Though, the relationship between self-perceived fitness might work via different pathways. On a speculative note, another explanation could be that the perception of one’s own fitness closely relates to the concept of self-efficacy and capability of acting. Therefore, these perceptions may share the same explaining variance which might partially eliminate the positive effect of self-efficacy and resilience to stress.

With mediation models we could verify evidence on the distinct associations between fitness and self-efficacy expectations on the one hand, and between self-efficacy and mental health on the other. Hereby, instead of fitness, previous studies mostly investigated the link between physical activity and self-efficacy [80, 81]. In this context it is worth noting that since we introduced both measures at baseline assessment, it is difficult to clearly assert about the effect’s direction. Utilising the social-cognitive theory by Bandura [82, 83] as a framework, activity levels and self-efficacy interact reciprocally. While physical activity has been found to contribute to heightened self-efficacy expectations, literature mostly supports the idea of self-efficacy to act positively on regular implementation and maintenance of activity or exercise participation [81, 84–86]. Only a few sports-related interventional studies have investigated self-efficacy as an outcome of sport involvement or exercise [87, 88]. Also in a recent review, sport scientists examined the potential mediating role of self-efficacy in the relationship between physical fitness and well-being. They could confirm that both fitness and self-efficacy play an important role for improved health, however, the causal relations remain unclarified [89]. This illustrates that more studies are needed to establish whether self-efficacy promotes physical activity or the other way around. Furthermore, we could show that higher self-efficacy was associated with lower reactivity to life events and daily stress, thus with proficient resilience outcomes. This is in line with earlier findings, demonstrating the beneficial effect of self-efficacy expectations on mental health and—in the context of positive appraisal style—on resilience to stress [45, 90, 91]. More specifically, this also confirms the potential stress buffering effect of self-efficacy in handling stressors and adversity, which was earlier tested in the context of daily stress, or traumatic events [41, 44, 92–94]. Altogether, the mediating effect of self-efficacy can be interpreted such as that higher levels of self-perceived and muscular fitness enforce one’s self-efficacy or mastery experience.

In this current work we restrained our analyses to a single, partly explaining psychosocial mechanism in the fitness–resilience relationship, whereby a full mediation model could not be obtained. This underlines the complexity of this relationship and indicates that various mechanisms may be involved. To entirely reveal the pathways underlying the relationship between fitness and resilience, psychophysiological mechanisms should also be considered. For example, according to the “Cross-Stressor Adaptation hypothesis (CSA)” evidence exists for regular exercise blunting hormonal and metabolic stressor reactivity of the hypothalamic–pituitary–adrenal (HPA) axis, due to a biological adaptation process [95]. Herein, lower stressor reactivity and faster stress recovery may explain the beneficial effects of exercise on mental health [31, 96, 97]. As we found that fitness seems to come along with reduced stressor reactivity, represented by less mental health problems, our findings can be considered to be in line with the CSA hypothesis. Matching this, self-efficacy has also been shown to impact the neuro-endocrinological stress response and seems to be negatively related to symptoms of distress after psychosocial stress [98]. Elsewhere, the positive effects of exercise have also been attributed to a reduction of excessive inflammation through oxidative stress [99–101] or enhanced neuroplasticity and growth factor expression inducing structural changes in the brain [102–105]. Especially in the context of resilience to stress, the potential mediating mechanisms of fitness on mental health mostly remain unclarified and should be considered.

### Limitations and recommendations for future research

Our results need to be interpreted in the light of several limitations. To the best of our knowledge, this present study is the first to investigate the association of physical fitness and stress reactivity in the form of mental health problems and, at the same time, to analyse the mediating role of self-efficacy on this relationship in a longitudinal study design. Therefore, it is difficult to compare the outcomes with earlier investigations that feature clear heterogeneity in methods and outcomes. In addition, for economic reasons, we opted for a composite score for muscular fitness, consisting of the well-validated hand strength test and the standing long jump. The standing long jump, as part of a fitness test battery, has already been applied in well-designed studies to capture physical fitness and health parameters [55, 106]. However, it should be considered that the standing long jump has mainly been used and validated in adolescents and is less frequently used in adults. Hence, the results concerning the standing long jump should be interpreted with caution.

Furthermore, due to their personal demographic specifications, the sample comprised of mostly young, well-educated and healthy, individuals. As the participants could participate the fitness assessment on a voluntary basis, self-selection bias is conceivable, which may account for a non-sufficient variation in participants’ fitness levels. Therefore, the results can hardly be generalised to individuals with low socioeconomic status as well as medical diagnoses and/or overweight. We recommend future studies to manage a greater differentiation in health status. Besides healthy individuals, considering clinical samples could increase generalizability and deliver distinguished information on psychologically burdened people. Conclusions regarding causality of the relationship between fitness and mediating self-efficacy are limited by the simultaneous assessment. In this respect, to be able to assert over the working direction, and changes over time due to fluctuations of the different measurements, we recommend methodological advanced cross-lagged panel designs, which could enable more insight into the reciprocal nature of physical activity, fitness, internal psychological resources, and mental health across time. From a methodological perspective, researchers and practitioners should be aware of the influence of stressor exposure when investigating the preventive effects of activity and fitness on mental health outcomes. From a preventive perspective, we cannot make a recommendation for specific intensities or durations of behavioural activations. However, the results allow for the recommendation of participation in physical activities that promote proficient muscular and self-perceived fitness. Since the maintenance of health-related activity over time may also depend on self-efficacy expectations [107], enhanced self-efficacy and mastery experience should play a considerable role in sport-based interventions for preventive purposes.

## Conclusion

Altogether, we could show that muscular as well as self-perceived fitness can be viewed as independent, prognostic parameters in relation to resilience to stress over a timespan of several months. A psychological mechanism underlying the association between muscular fitness and stress resilience seems to comprise self-efficacy expectations. Therefore, our study supports the notion that fitness and resulting proficient self-efficacy expectations may have complementary benefits for the promotion of mental health.

## Data Availability

Data can be made available by the author upon request.
